# A SELDI mass spectrometry study of experimental autoimmune encephalomyelitis: sample preparation, reproducibility, and differential protein expression patterns

**DOI:** 10.1186/1477-5956-11-19

**Published:** 2013-05-01

**Authors:** Sausan Azzam, Laurie Broadwater, Shuo Li, Ernest J Freeman, Jennifer McDonough, Roger B Gregory

**Affiliations:** 1 Department of Chemistry and Biochemistry, Kent State University, Kent, Ohio 44242, USA; 2 Department of Biological Sciences, Kent State University, Kent, Ohio 44242, USA

## Abstract

**Background:**

Experimental autoimmune encephalomyelitis (EAE) is an autoimmune, inflammatory disease of the central nervous system that is widely used as a model of multiple sclerosis (MS). Mitochondrial dysfunction appears to play a role in the development of neuropathology in MS and may also play a role in disease pathology in EAE. Here, surface enhanced laser desorption ionization mass spectrometry (SELDI-MS) has been employed to obtain protein expression profiles from mitochondrially enriched fractions derived from EAE and control mouse brain. To gain insight into experimental variation, the reproducibility of sub-cellular fractionation, anion exchange fractionation as well as spot-to-spot and chip-to-chip variation using pooled samples from brain tissue was examined.

**Results:**

Variability of SELDI mass spectral peak intensities indicates a coefficient of variation (CV) of 15.6% and 17.6% between spots on a given chip and between different chips, respectively. Thinly slicing tissue prior to homogenization with a rotor homogenizer showed better reproducibility (CV = 17.0%) than homogenization of blocks of brain tissue with a Teflon® pestle (CV = 27.0%). Fractionation of proteins with anion exchange beads prior to SELDI-MS analysis gave overall CV values from 16.1% to 18.6%. SELDI mass spectra of mitochondrial fractions obtained from brain tissue from EAE mice and controls displayed 39 differentially expressed proteins (p≤ 0.05) out of a total of 241 protein peaks observed in anion exchange fractions. Hierarchical clustering analysis showed that protein fractions from EAE animals with severe disability clearly segregated from controls. Several components of electron transport chain complexes (cytochrome c oxidase subunit 6b1, subunit 6C, and subunit 4; NADH dehydrogenase flavoprotein 3, alpha subcomplex subunit 2, Fe-S protein 4, and Fe-S protein 6; and ATP synthase subunit e) were identified as possible differentially expressed proteins. Myelin Basic Protein isoform 8 (MBP8) (14.2 kDa) levels were lower in EAE samples with advanced disease relative to controls, while an MBP fragment (12. 4kDa), likely due to calpain digestion, was increased in EAE relative to controls. The appearance of MBP in mitochondrially enriched fractions is due to tissue freezing and storage, as MBP was not found associated with mitochondria obtained from fresh tissue.

**Conclusions:**

SELDI mass spectrometry can be employed to explore the proteome of a complex tissue (brain) and obtain protein profiles of differentially expressed proteins from protein fractions. Appropriate homogenization protocols and protein fractionation using anion exchange beads can be employed to reduce sample complexity without introducing significant additional variation into the SELDI mass spectra beyond that inherent in the SELDI- MS method itself. SELDI-MS coupled with principal component analysis and hierarchical cluster analysis provides protein patterns that can clearly distinguish the disease state from controls. However, identification of individual differentially expressed proteins requires a separate purification of the proteins of interest by polyacrylamide electrophoresis prior to trypsin digestion and peptide mass fingerprint analysis, and unambiguous identification of differentially expressed proteins can be difficult if protein bands consist of several proteins with similar molecular weights.

## Background

Multiple sclerosis (MS) is an inflammatory neurodegenerative disease of the central nervous system (CNS) characterized by demyelination, oligodendrocyte loss, axonal damage and neurodegeneration which results in progressive physical and cognitive disability [1]. Experimental autoimmune encephalomyelitis (EAE) is an autoimmune, inflammatory disease of the central nervous system that mimics many of the clinical and histological features of MS, including the presence of cellular infiltrates as well as demyelination and axonal degeneration in the CNS [2,3]. As a result, EAE has been widely employed as a model for studying the pathogenesis of MS and for the development of therapeutic approaches to treat the disease [4-8]. There is much interest in the role of oxidative stress and mitochondrial dysfunction in human disease including cardiovascular disease and neurodegenerative diseases [9-12]. A number of studies implicate mitochondrial dysfunction in the development of neuropathology in MS. Decreases in the neuronal mitochondrial metabolite N-acetyl aspartate (NAA) in MS brain have been observed by nuclear magnetic resonance spectroscopy and a decrease in NAA appears to precede neuronal atrophy, suggesting that mitochondrial dysfunction may precede neurodegeneration [13,14]. Alterations to mitochondrial enzyme activity and damage to mitochondrial DNA have been observed in MS white matter lesions [16-20]. In addition, defects in mitochondrial electron transport gene expression and function in normal appearing gray matter (NAGM) in postmortem MS cortex have been reported [21,22]. Mitochondrial dysfunction may also play a role in disease pathology in EAE. Increased nitration of components of the electron transport chain leads to decreased mitochondrial activity in EAE [15].

Proteomic profiling has been successfully employed in the discovery and identification of biomarkers in neurodegenerative diseases [23,24] and in the analysis of mitochondrial proteomes in disease [12]. A number of studies utilizing proteomic approaches to investigate the differentially expressed proteins in MS and EAE have been reported [25-33]. Proteomic profiling is a rapidly developing technology that may provide clues to the mechanisms underlying the onset and progression of these diseases. A variety of approaches are available for the analysis of proteomes, including 2D polyacrylamide gel electrophoresis (2D-PAGE) and Differential Gel Electrophoresis (DIGE) [34-36], liquid chromatography coupled to high resolution mass spectrometry (LC-MS), where label-free [37] as well as ICAT [38] and iTRAQ [39] labeling strategies are available for quantitative work and differential analysis [40], and Surface Enhanced Laser Desorption/Ionization Mass Spectrometry (SELDI-MS) [41-44].

SELDI-MS was originally developed for rapid, high throughput biomarker discovery in biological fluids (plasma, serum, urine, cerebrospinal fluid) but has been employed to examine the proteomes of cell lysates and tissues [43,45]. The approach combines time-of-flight mass spectrometry with protein capture using a variety of chromatographic protein chip surfaces (anion and cation exchange, normal phase, reverse phase, and immobilized metal ion) as well as chemistries for covalent attachment of proteins for affinity capture. It is most sensitive for monitoring low molecular weight (< 20kDa) proteins and peptides. Complex protein samples may also be fractionated (i.e. fractionation on anion exchange beads) prior to spotting protein chips in order to reduce the complexity of SELDI mass spectra. SELDI-MS Expression Difference Mapping allows rapid analysis of multiple samples over multiple conditions to identify differentially expressed proteins. Principal component analysis (PCA) and hierarchical clustering can also be employed to identify the mass spectral peaks that distinguish disease from controls [46-51]. Reproducibility in various proteome profiling technologies, including SELDI-TOF mass spectrometry, has been a major challenge. Sources of variability intrinsic to the SELDI-MS technique include variation in the ionization and desorption processes and in time-of-flight measurement and ion detection. Errors also can be introduced in mass spectral preprocessing steps such as baseline correction, normalization, and mass spectra alignment. Attempts to reduce proteome complexity and enhance protein identification by the use of sample fractionation steps, including sub-cellular and anion exchange fractionation, can also introduce variability from sample to sample.

In this study, we used SELDI-TOF-MS to obtain protein profiles from mitochondrially-enriched fractions derived from EAE and control mouse brain. To gain insight into sources of experimental variability, we examined the reproducibility of sub-cellular fractionation, anion exchange fractionation as well as spot-to-spot and chip-to-chip variation using pooled samples from brain tissue.

## Results

### Spot-to-spot and chip-to-chip variability

**Figure 1:**
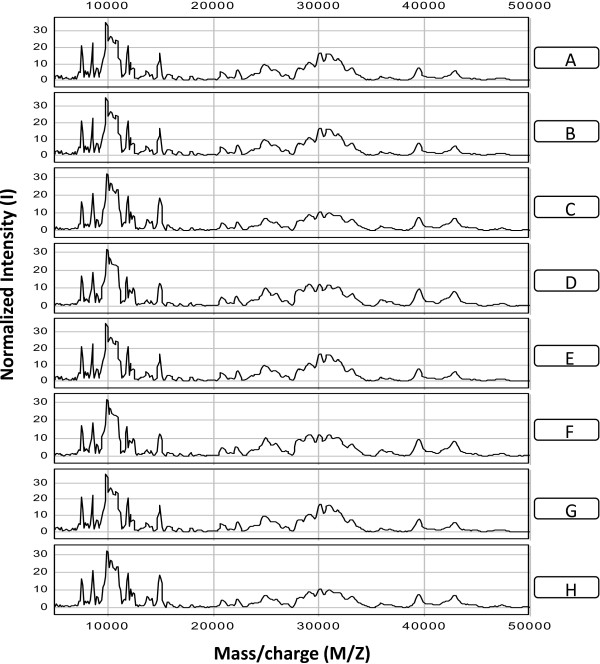
**Reproducibility of SELDI-TOF-MS spectra.** Examples of SELDI mass spectra obtained for samples of anion exchange fraction Q1 from the cytosolic fraction of pooled mouse brain tissue spotted in eight replicates (A-H) onto an NP20 Protein Chip.

In order to obtain a measure of the reproducibility of the SELDI mass spectral analysis, equal volumes (1.0 μL) of anion exchange fraction Q1 from the cytosolic fraction of pooled mouse brain tissue was spotted onto four NP20 protein chips (eight spots per chip) allowing an analysis of both spot-to-spot variation within a chip as well as chip-to-chip variation. The SELDI-MS spectra of each spot (A-H) obtained for one NP20 protein chip is shown in Figure 1. A total of eighteen peaks with signal/noise (S/N) > 5 were found to be common to all spectra measured on this chip which represented proteins with m/z ratios in the range from 7,500 to 42,772 Da. All spectra have very similar peak patterns as expected for replicates from the same pooled sample. Sources of variability include variation in manually spotting proteins on the chip, the reproducibility of the manufactured spot surfaces, and the intrinsic variation in the ionization and desorption process and in ion time-of-flight and detection. The coefficient of variation for each peak is listed in Table 1. The overall coefficient of variation for the peak intensity for this protein chip was 17.3%. No significant differences in the variability of peak intensities were observed as a function of mass/charge ratio. Analysis of the other three NP20 chips gave similar results with overall coefficients of variation for the peak intensities of 13.6%, 16.1%, and 15.3%, suggesting an average coefficient of variation for spot-to-spot variability of 15.6%. A similar analysis of the chip-to-chip variability of peak intensities suggests a slightly larger overall coefficient of variation of 17.6%. These values are similar to those reported by others for analyses performed on single machines [52-54].

**Table 1:** Spot-to-spot variability of SELDI-TOF mass spectra

		**Peak Intensity**			**Peak M/Z**	
**Peak**	**Mean**	**STD**	**CV%**	**Mean**	**STD**	**CV%**
1	16.0	2.4	15.3	7496.3	2.6	0.03
2	18.5	3.2	17.1	8558.0	2.3	0.03
3	28.5	3.9	14.0	9900.3	3.3	0.03
4	21.2	5.8	27.2	10254.0	1.9	0.02
5	20.8	2.7	13.2	10860.5	2.6	0.02
6	16.8	4.1	24.7	11819.5	3.6	0.03
7	10.9	3.2	29.5	12120.4	3.9	0.03
8	9.1	1.8	20.2	12360.3	3.8	0.03
9	4.1	0.6	13.8	20791.1	16.9	0.08
10	4.8	0.6	12.5	22253.6	16.3	0.07
11	7.4	1.3	17.4	24904.4	18.0	0.07
12	6.0	1.5	24.8	28150.4	18.0	0.07
13	9.1	1.0	11.5	29033.2	25.3	0.09
14	12.0	2.1	17.7	30038.2	11.6	0.04
15	11.6	2.1	17.9	30893.0	9.2	0.03
16	6.3	0.6	10.1	33025.8	24.7	0.07
17	6.8	0.7	10.0	39397.1	21.8	0.06
18	6.0	0.8	13.9	42781.4	20.9	0.05

The mean, standard deviation, and coefficient of variation (CV%) for eighteen protein peaks from an ion exchange fraction (Q1) of the cytosolic fraction of pooled mouse brain tissue. The overall CV% for peak intensity and m/z ratio on this chip were 17.3% and 0.05%, respectively.

### Reproducibility of homogenization and subcellular fractionation protocols

Depletion of high abundance proteins and fractionation of samples to reduce proteome complexity prior to SELDI-TOF MS analysis can increase the number of detected peaks but can introduce additional variability into the analysis beyond that associated with the SELDI MS method itself [55,56]. In order to focus on possible differences in the mitochondrial proteome, to reduce the proteome complexity of brain tissue, and increase the number of detected peaks, particularly for low abundance proteins, we employed sub-cellular fractionation as well as anion exchange fractionation. Our recent work on the proteomics of Multiple Sclerosis has focused on changes occurring in normal appearing grey matter (NAGM) rather than in whole brain or white matter tissue that might include lesions. This type of analysis requires a tissue preparation protocol in which brain tissue is cut into thin slices and the slices flanking the tissue to be homogenized are examined under a microscope to confirm the absence of lesions prior to homogenization.

We have therefore compared the reproducibility of two homogenization methods: method M1, in which tissue was thinly sliced (60 μm, ~ 250 mg) prior to homogenization with a rotor homogenizer (Brinkmann blender), and method M2, in which blocks (~250 mg) of brain tissue were cut and homogenized with a mechanical homogenizer (Teflon® pestle). To conduct the analysis, pooled mouse brain tissue (n=6) was fractionated into enriched nuclear, cytosolic, and mitochondrial fractions. Three trials were conducted for each homogenization method.

**Figure 2:**
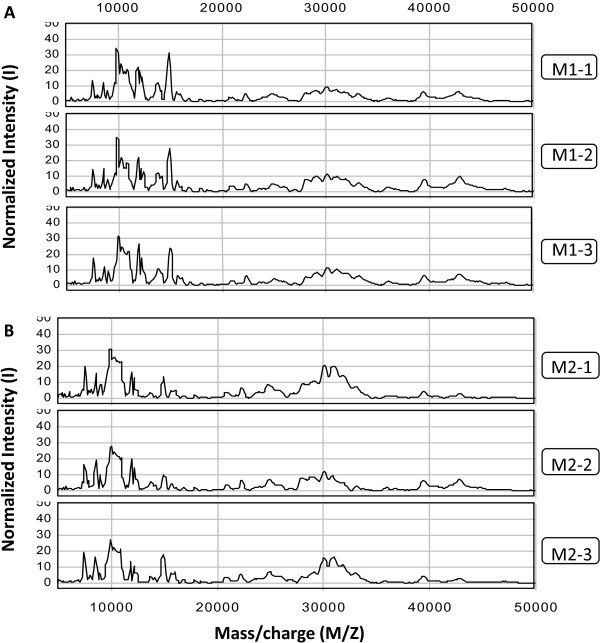
**Reproducibility of subcellular fractionation.** Examples of SELDI mass spectra obtained for three replicates of the anion exchange fraction Q1 from the cytosolic fraction of pooled mouse cytosolic homogenized using method M1 (top) and method M2 (bottom).

The cytosolic fraction was spotted as four replicates onto NP20 chips. Examples of SELDI mass spectra for the cytosolic Q1 fraction from each trial obtained with the two homogenization methods are shown in Figure 2. Fourteen peaks with S/N >5 were found to be common to all mass spectra analyzed across the two homogenization protocols and represent proteins with mass/charge ratios from 7,492 Da to 42,764 Da. The coefficients of variation for each peak are listed in Table 2 and varied from 8.2% to as much as 60.7%. Overall, homogenization method M1, in which tissue was thinly sliced prior to homogenization with a rotor homogenizer, shows better reproducibility than method M2, in which blocks of brain tissue were cut and homogenized with a mechanical homogenizer.

**Table 2:** Reproducibility of homogenization and subcellular fractionation protocols

	**M1**	**M2**
**M/Z**	**CV%**	**CV%**
7487	16.8	20.1
10854	20.0	16.2
11814	8.2	20.5
12106	23.1	42.7
15615	45.4	60.7
20758	8.5	18.7
24905	14.7	38.4
28979	11.4	17.0
30002	14.0	32.3
30818	8.6	35.5
31718	20.2	27.0
33018	28.3	19.5
39336	8.9	7.1
42760	10.0	22.1
**Overall CV%**	**17**	**27**

The coefficient of variation (CV%) for fourteen protein peaks from an ion exchange fraction (Q1) of the cytosolic fraction of pooled mouse brain tissue obtained for two homogenization protocols in which tissue was either thinly sliced prior to homogenization with a Brinkmann rotor homogenizer (method M1) or tissue was cut into ~250 mg blocks and homogenized with a Teflon pestle (method M2). The overall CV% for the peak intensities was 17.0% for method M1 and 27% for method M2.

The overall coefficient of variation for method M1 was 17.0% compared with 27.0% for method M2.

The two methods gave a similar pattern of protein intensities, and significant differences in mean peak intensities with the two homogenization methods were observed only for one protein peak (m/z = 11,814 Da). Nevertheless, it is clear that method M2 introduces significant additional variability beyond that contributed by the SELDI-MS method itself, while homogenization method M1 together with the subcellular fractionation method employed here yield highly reproducible protein samples for proteomic analysis.

### Reproducibility of anion exchange fractionation

The use of anion exchange beads to fractionate samples prior to SELDI-MS analysis greatly improves the resulting mass spectra, but the procedure must be highly reproducible in order to avoid differences in protein retention on the beads or release from the beads, which would alter protein profiles of the fractions from sample to sample. To this end, buffer concentrations and pH must be carefully monitored and, ideally, anion exchange fractionation performed on all samples on the same day, as was the case with this analysis. Anion exchange of cytosolic fractions with BioSepra Q Ceramic HyperD anion exchange beads yielded six fractions (Q1– Q6). Three trials were performed and three fractions (Q1, Q2, and Q4) from each trial were spotted in triplicate onto NP20 protein chips. Seven peaks with S/N > 5 were found that were common to all SELDI mass spectra of fraction Q1. The overall coefficient of variation for the peak intensities was 16.1%. Analyses of fraction Q2, with 11 peaks common to all spectra, and Q4, with 8 peaks common to all spectra, gave overall coefficients of variation of 18.6% and 17.3% for peak intensity, respectively.

These results suggest that anion exchange pre-fractionation can be performed reproducibly and does not introduce significant additional variation to the SELDI mass spectra beyond that inherent in the SELDI-MS method itself, which is comparable to the variability reported by other SELDI-MS users [52,54].

### EAE protein expression profiles

The clinical appearance of EAE is of an ascending myelitis, the severity of which is scored on a scale from 0 (normal animal) to 5 (moribund state). SELDI mass spectra were determined for mitochondrial fractions obtained from brain tissue from one mouse (labeled E1) at EAE disease stage 1 (animals display tail paralysis with mild meningeal inflammation), and four mice (labeled E2 – E5) at EAE disease stage 3 (animals display complete paralysis of one or both hind limbs with severe meningitis, and parenchyma infiltration with multiple perivascular infiltration), as well as from brain tissue from three controls (labeled C1 – C3). The mitochondrial subcellular fractions were further fractionated on anion exchange beads to give six fractions (Q1- Q6) for SELDI-MS analysis. A total of 241 protein peaks (clusters) were identified in these six fractions which met our selection criteria. A total of 39 protein peaks were found to be differentially expressed (p≤ 0.05) (see Table 3). Eleven proteins were found at greater levels in EAE brains relative to controls and 28 proteins were found at lower levels in EAE brains relative to controls. As shown in Table 3, the differentially expressed proteins are seen in all six anion exchange fractions (Q1-Q6) and include proteins with mass/charge ratios from 3,006 to 49,085 Da. This represents a large fraction (16%) of the observed peaks, and it is possible that some proteins have been counted twice (i.e. they are observed under both the LMW and HMW acquisition conditions employed here), for example, the protein peaks at mass/charge ratios of 13,365 (HMW) and 13,331 (LMW) in fraction Q2; 12,448 (HMW) and 12,493 (LMW) in fraction Q3; 16,910 (HMW) and 16,878 (LMW) in fraction Q5; and 13,854 (HMW) and 13,809 (LMW) in fraction Q6. In addition, some peaks with different m/z ratios may represent proteolytic fragments of the same protein.

**Table 3:** Differentially expressed protein peaks in EAE as compared to control brain tissue

**Fraction (Q)**	**Mass (m/z)**	**Acquisition settings**	**p-Value**	**ROC Area**	**Expression change in EAE**
1	108995	HMW	0.03	0.00	↓
2	6540	HMW	0.05	0.13	↓
2	10274	HMW	0.05	0.07	↓
2	11691	HMW	0.05	1.00	↑
2	13365	HMW	0.05	0.07	↓
2	14349	HMW	0.05	1.00	↑
2	16836	HMW	0.05	0.00	↓
2	31521	HMW	0.05	0.07	↓
2	13331	LMW	0.03	0.00	↓
2	49085	LMW	0.05	0.06	↓
3	8044	HMW	0.02	0.00	↓
3	10416	HMW	0.03	0.07	↓
3	10862	HMW	0.02	0.00	↑
3	12448	HMW	0.05	0.96	↑
3	13954	HMW	0.05	0.03	↓
3	8251	LMW	0.05	0.00	↓
3	10855	LMW	0.03	0.00	↓
3	12493	LMW	0.05	0.95	↑
3	15498	LMW	0.03	0.00	↓
4	9841	HMW	0.03	1.00	↑
4	42924	HMW	0.05	0.93	↑
4	6583	LMW	0.05	0.07	↓
4	9115	LMW	0.05	0.07	↓
5	8528	HMW	0.02	0.00	↓
5	16910	HMW	0.02	0.00	↓
5	17307	HMW	0.02	0.00	↓
5	8392	LMW	0.03	0.00	↓
5	9801	LMW	0.05	0.93	↑
5	16878	LMW	0.03	0.00	↓
5	17280	LMW	0.05	0.00	↓
6	9717	HMW	0.03	0.00	↓
6	13854	HMW	0.03	1.00	↑
6	3006	LMW	0.03	0.00	↓
6	3266	LMW	0.03	0.00	↓
6	4272	LMW	0.03	0.00	↓
6	5455	LMW	0.03	0.00	↓
6	10637	LMW	0.05	0.07	↓
6	13809	LMW	0.03	1.00	↑
6	23228	LMW	0.05	0.93	↑

Fraction Q: number of the ion exchange fractionation; m/z: mass/charge ratio of the protein peak; LMW: low molecular weight protocol; HMW: high molecular weight protocol; ↑: protein level was increased in EAE as compared to control; ↓: protein level was decreased in EAE as compared to control.

Protein expression profiles were further analyzed with hierarchical clustering techniques [49] and principal component analysis (PCA) [46,47,57]. Hierarchical clustering of differentially expressed proteins employed a modified Pearson product-moment correlation coefficient as a distance measure and used the average linkage method to compute dendrograms in which the nodes join “objects” (protein fractions) with the most similar protein expression profiles. The relative length of the branches indicates the similarity of the expression profiles.

**Figure 3:**
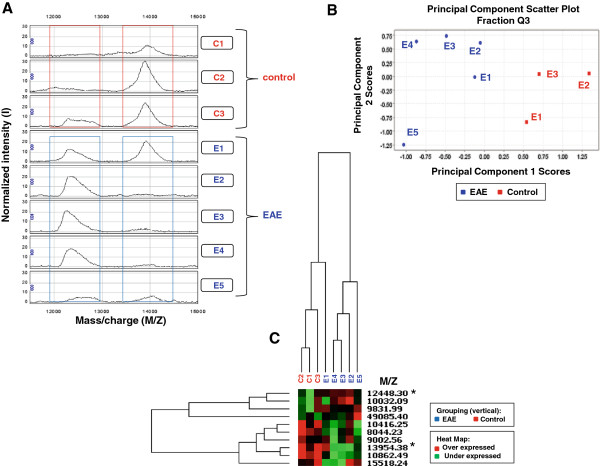
**Protein expression profile for fraction Q3. (A)** Example of SELDI mass spectra of two differentially expressed proteins in anion exchange fraction Q3 obtained from mitochondrial fractions of brain tissue from EAE mice at disease stage 1 (E1) and disease stage 3 (E2-E5) as well as brain tissue from controls (C1-C3). The peak at 12448 Da shows increased expression in EAE tissue relative to controls, whereas the peak at 13954 Da shows a decreased expression in EAE as compared to controls. **(B)** Scatter plot of scores for principal components 1 and 2 obtained from a PCA analysis showing the segregation of data into EAE and control samples. **(C)** Heat map obtained from a hierarchical cluster analysis showing the clustering of the E1 (disease stage 1) sample with the control samples. The * indicates the two differentially expressed proteins shown in the SELDI mass spectra given in **(A).** These two proteins were digested with trypsin and identified by peptide mass fingerprinting and peptide sequencing with a LTQ-FT mass spectrometer (Table 4).

**Figure 4:**
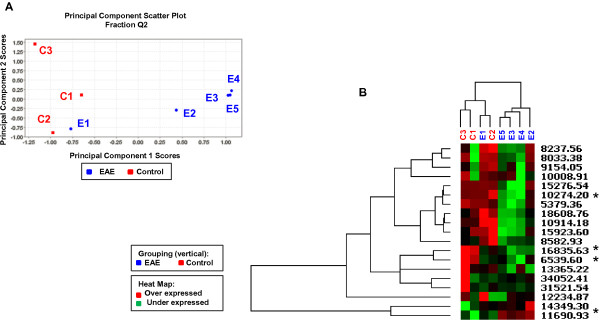
**Protein expression profile for fraction Q2. (A)** Scatter plot of scores for principal components 1 and 2 obtained from a PCA analysis showing the segregation of data into EAE (blue) and controls (red). **(B)** Hierarchical clustering resulted in two major clusters: EAE stage 3 samples (E2-E5) and control (C1-C3) + EAE stage 1 (E1) samples. The * indicates the four differentially expressed proteins that were subjected to trypsin digestion and identified by peptide mass fingerprinting and peptide sequencing with a LTQ-FT mass spectrometer.

The result of a hierarchical clustering analysis of fraction Q3 obtained with a HMW data acquisition protocol is given in Figure 3C and shows that the protein fractions are segregated into two distinct groups: (EAE stage 3) and (controls + EAE stage 1). This clustering of the EAE disease stage 1 sample with controls was also observed with fraction Q2 (Figure 4B). However, the segregation of EAE disease stage 1 with controls may not be significant as only one sample at this disease stage was examined.

Principal component analysis allows us to explore patterns and identify the most important sources of variation in large datasets. Principal component analysis computes a set of orthogonal directions called principal components. The first principal component is the eigenvector of the covariance matrix which has the largest eigenvalue and accounts for the greatest variation in the data. The second principal component (i.e. with the next largest eigenvalue) is the eigenvector, orthogonal to the first principal component, which accounts for the next most variance, and so on. The contribution of each principal component to a particular protein expression profile is given by the principal component score. The first several principal components often account for much of the variability in the data and indicate which features most contribute to this variability, while the principal component scores relate that variability to each sample.

**Figure 5:**
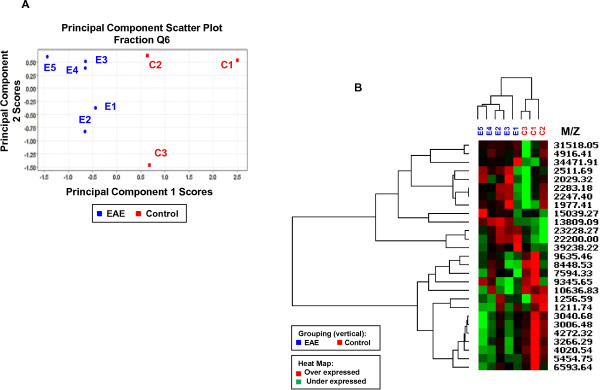
**Protein expression profile for fraction Q6. (A)** Scatter plot of scores for principal components 1 and 2 obtained from a PCA analysis showing the segregation of data into EAE and control samples. **(B)** Heat map obtained from a hierarchical cluster analysis showing the clustering of EAE and control samples into distinct groups. Note also the highly correlated peaks at 3006, 3040, 3266, 4020, and 4272 Da in the lowest node of the heat map suggesting a strong relationship between the peaks.

As a result, the essential features of the data can often be captured in a model of much smaller dimensionality than the original dataset. In the case of the Q3 HMW dataset, the first three principal components account for ~84% of the variability in the data. A scatter plot (Figure 3B) of the scores for principal components 1 and 2 clearly shows the segregation of the dataset into EAE and control samples. Analysis of other fractions shows a similar behavior. For example, the scatter plot of scores for principal components 1 and 2 obtained from a Principal Component Analysis of fraction Q6 with a LMW acquisition protocol (Figure 5) also shows a clear segregation of EAE and control samples. Hierarchical clustering analysis can help identify interesting correlations among peaks. For example, the lowest node in the dendrogram for fraction Q6 LMW consists of five highly correlated peaks at 3006, 3040, 3266, 4020, and 4272 Da suggesting a strong relationship between the peaks. Three of them are differentially expressed with p values of 0.03. Without further analysis, the basis of this correlation cannot be determined, but strongly correlated peaks in this mass range could reflect proteolytic fragments of the same protein, the expression of a set of related proteins in response to a stimulus, or post-translationally modified species.

Principal component analysis of fraction Q2 with a LMW acquisition protocol (Figure 4) shows a clear discrimination of EAE stage 3 samples from controls + EAE stage 1 samples. Hierarchical clustering analysis also clusters the EAE stage 1 (E1) sample with controls rather with the more advanced EAE disease samples (E2 – E5). The possibility that some protein fractions might be able to discriminate early from advanced EAE disease is interesting. However, with only one early stage EAE sample, this clustering may not be significant. In all cases, the first three principal components account for most (>80%) of the variation in the data, indicating that much of the structure in the dataset can be represented by a relatively low dimensionality analysis.

### Identification of differentially expressed proteins

SELDI-MS workflows enable the rapid analysis of a large numbers of protein samples and can generate patterns of differentially expressed proteins that may distinguish disease states from controls. However, protein identification usually requires separate purification of the proteins of interest by polyacrylamide gel electrophoresis prior to trypsin digestion and peptide mass fingerprint analysis, and confirmation of protein identity by Western blotting analysis. Unambiguous identification of differentially expressed proteins can be difficult if protein bands obtained from 1D polyacrylamide gel electrophoresis (PAGE) consist of several proteins with similar molecular weights. However, the subcellular fractionation of the sample into cytosolic, mitochondrial, and nuclear fractions combined with ion exchange fractionation prior to 1D PAGE employed in the current work is actually a multi-dimensional separation, albeit with low resolution fractionations (3 subcellular fractions and 6 ion exchange fractions) for the first two steps, and one might expect more success in resolving individual proteins than with 1D PAGE alone.

Fractions Q2, Q3, Q4, and Q5 from control or EAE brain tissue were subjected to 1D SDS polyacrylamide gel electrophoresis together with SeeBlue Plus 2 pre-stained molecular weight standards. The gels were stained with Coomassie Brilliant blue R 250 and destained overnight.

Protein bands corresponding to the molecular weights of differentially expressed proteins of interest were excised and the proteins in the gel plugs were treated with dithiothreitol and iodoacetamide to reduce any disulfide bonds and alkylate the resulting thiol groups. The proteins were digested with trypsin and identified by peptide mass fingerprinting and peptide sequencing with a LTQ-FT mass spectrometer at the Center for Proteomics and Bioinformatics, Case Western Reserve University. The resulting peak list files were used to interrogate the indexed IPI mouse database with the Mascot algorithm. The list of identified proteins is given in Table 4 and is dominated by components of the electron transport chain including cytochrome c oxidase subunit 6b1 (COX6b1), subunit 6C (COX6C), and subunit 4 (COX4i1); NADH dehydrogenase flavoprotein 3 (Ndufv3), alpha subcomplex subunit 2 (Ndufa2), Fe-S protein 4 (Ndufs4), and Fe-S protein 6 (Ndufs6); and ATP synthase subunit e (Atp5k); as well as myelin basic protein isoforms. Other proteins include ubiquitin A-52 protein (Uba52), a protein involved in ion homeostasis; CPN10-like protein Hspe-rs1 (Cpn10-rs1), which is a member of the GroES chaperonin family; beta2 microglobulin (B2m); cofilin 1 (Cfl1); V-type proton ATPase subunit G2 (Atp6v1g2), a vacuolar ATPase which mediates the acidification of intracellular compartments; basic transcription factor 3 (btf3) and the 40S ribosomal protein S13 (Rps13). Because the protein bands in fraction Q3 each consist of a single identified protein, the identity of the differentially expressed proteins as myelin basic protein (MBP) isoforms is unambiguous. However, we have not confirmed which of the other proteins identified in gel bands represents the differentially expressed protein peaks observed in SELDI mass spectra. Recent studies have implicated mitochondrial dysfunction as a possible mechanism in the development of neuropathology in MS (13, 14, 21, 22, 31, and 58) and increased levels of nitration among proteins of the electron transport chain result in reduced mitochondrial activity in EAE [59].

**Table 4:** Protein identification

**Fraction (Q)**	**Mass (m/z)**	**p-Value**	**Accession number**	**Entry name**	**Theoretical MW**	**MASCOT score**	**Peptides detected**	**Sequence coverage**
**2**	6550	0.05	IPI00138892	Uba52	14719	642	7	46
			IPI00225390	COX6b1	10065	84	2	24
	10274	0.05	IPI00225390	COX6b1	10065	545	7	67
			IPI00120045	Hspe1-rs1	10971	431	3	33
			IPI00403381	Ndufv3	11806	430	4	36
	11691	0.05	IPI00109966	B2m	13814	499	4	27
			IPI00128345	Ndufs6	13012	414	3	31
	16836	0.05	IPI00890117	CfI1	18548	663	12	70
			IPI00229008	Ndufs4	19772	335	8	48
**3**	12448	0.05	IPI00115240	MBP1	27151	443	5	15
	13954	0.05	IPI00223381	MBP8	14202	584	12	61
**4**	9841	0.03	IPI00131771	COX6C	8464	1460	9	63
			IPI00223593	MBP10	20801	1049	11	50
			IPI00111770	Atp5k	8230	473	7	68
			IPI00225390	COX6b1	10065	443	6	53
			IPI00315302	Ndufa2	10909	428	5	41
**5**	16910	0.02	IPI00223379	MBP6	17215	2521	14	64
			IPI00123817	Atp6v1g2	13643	501	4	39
	17307	0.02	IPI00223380	MBP7	17230	1168	12	70
			IPI00319231	Rps13	17212	362	7	45
			IPI00229008	Ndufs4	19772	354	8	34
			IPI00131186	Btf3	17688	329	3	46

Proteins identified in 1D polyacrylamide gel bands which correspond to the molecular weights of differentially expressed proteins in EAE relative to controls found by SELDI-MS analysis. Only the myelin basic protein isoforms found in fraction Q3 are unambiguously identified.

The appearance of a number of components of electron transport chain complexes (cytochrome c oxidase subunit 6b1, subunit 6C, and subunit 4; NADH dehydrogenase flavoprotein 3, alpha subcomplex subunit 2, Fe-S protein 4, and Fe-S protein 6; and ATP synthase subunit e) among the list of potential differentially expressed proteins is interesting given the identification of cytochrome c oxidase subunit 4 (32), subunit 5a (30), and subunit 5b (30, 31) as being differentially expressed in EAE or MS in previous studies. A recent proteomics study of spinal cord during the clinical course of EAE (33) identified 35 differentially expressed proteins out of 800 spots observed by 2D PAGE, including proteins involved in energy pathways and cell growth, and transport processes. Only a few mitochondrial proteins were differentially expressed, and none correspond to the mitochondrial proteins described here. Farias et al (33) found decreased expression of vacuolar ATPase subunit B in EAE relative to controls, while we observe vacuolar ATPase subunit G2 (Atp6v1g2) in differentially expressed band Q5. This V-type proton ATPase mediates the acidification of intracellular compartments.

### Myelin basic protein

**Figure 6:**
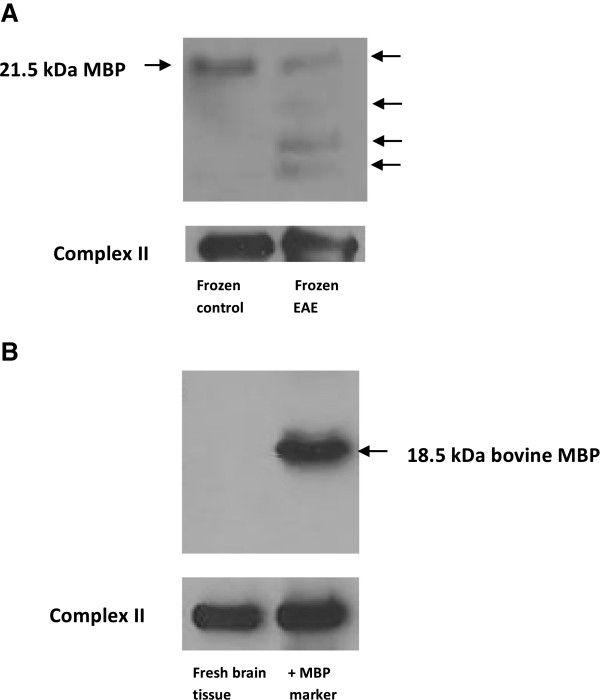
**MBP is not observed in mitochondrial fractions from fresh brain tissue.** Western blot showing **(A)** the presence of MBP in a mitochondrial fraction derived from frozen control (left) and frozen EAE (right) mouse brain and **(B)** the absence of MBP in mitochondrial fractions derived from fresh mouse brain tissue (left) together with a positive control prepared by adding purified bovine 18.6 kDa MBP isoform to the mitochondrial fraction derived from fresh mouse tissue (right).

The identity of the differentially expressed proteins in fraction Q3 as myelin basic protein isoforms was confirmed with Western blots (not shown). MBP is not expected to be associated with mitochondria. Indeed Ravera et al [60] reported finding no MBP in mitochondria using fresh bovine brain tissue and slightly different preparation conditions. MBP is a basic protein that interacts with membranes and it is possible that the mitochondrially-enriched fraction was contaminated with MBP released from myelin during tissue homogenization. To test this we added fluorescently labeled MBP to brain tissue prior to homogenization and measured the fluorescence intensity of nuclear, mitochondrial and cytoplasmic fractions. Most fluorescence is observed in the cytoplasmic (70%) and nuclear (22%) fractions, with less than 2% of the total fluorescence observed in the mitochondrially enriched fraction. While this is only a small fraction of the total MBP added, we cannot rule out contamination of the mitochondrial fraction by free MBP. Freezing and storage are known to lead to contamination of mitochondria by synaptosomal membrane fragments [61] and it is possible that freezing and storage of brain tissue blocks prior to homogenization and subcellular fractionation also leads to contamination of mitochondria by myelin membrane fragments or myelin vesicles. To test this we employed Western blots to determine if contamination was also observed in the mitochondrial fraction obtained with fresh mouse brain tissue. The presence of MBP isoforms in mitochondrial fractions from frozen control and EAE mouse brain tissue was confirmed in a Western blot probed with antibodies to mouse MBP (Figure 6A). The 21.5 kDa MBP isoform was clearly seen in both frozen control and EAE mouse samples. In addition, other MBP isoforms or MBP fragments are observed in the frozen EAE sample. By contrast, no MBP was detected in mitochondrial fractions derived from fresh mouse tissue (Figure 6B left lane). The positive control (Figure 6B right lane) was prepared by adding purified bovine 18.6 kDa MBP isoform to the mitochondrial fraction derived from fresh mouse tissue. This gel was probed with both mouse and bovine anti-MBP antibodies.

So it appears that the presence of MBP in the mitochondrial fraction is an artifact of tissue freezing and storage. Nevertheless, differential expression of MBP isoforms and proteolytic fragments appears to be a consistent feature of EAE. Increases in expression of MBP isoforms also have been observed in mitochondrially-enriched fractions from MS brain tissue relative to controls, where the differential expression of MBP was an important factor separating MS samples from controls in principal component analysis [31]. We have identified the peak at 13,954 Da, which decreases in EAE relative to controls, as MBP8 (the 14.2 kDa isoform). There is also a peak at 12,448 Da, which increases in EAE relative to controls, and is identified as MBP1, the canonical MBP, but in fact, the relatively small number of peptides (five) and sequence coverage (15%), mean this peak could be any MBP isoform except isoform 3. Its m/z ratio is consistent with it being a proteolytic fragment of MBP. The reduction of the 14.2 kDa MBP isoform and the increase in the 12.4 kDa MBP fragment in EAE animals with stage 3 diseases is clearly evident in the SELDI mass spectra shown in Figure 3A. Proteolysis has long been thought to play a role in multiple sclerosis, and elevated proteolytic activity has been observed in cerebrospinal fluid in MS patients [62]. Several proteases have been implicated in the degradation of myelin proteins and the generation of immunogenic peptides in multiple sclerosis and experimental autoimmune encephalomyelitis (EAE) including calpain, trypsin 4, matrix metalloproteinase, myelencephalon-specific protease (MSP), plasminogen activators and cathepsin D [63-66]. Liu et al. [65] reported fragmentation of MBP following traumatic brain injury, and linked this to the protease calpain with a major cleavage site between Phe-and Lys of the peptide TQDENPVVHFF-–K. They reported the degradation of the 21.5 and 18.5kDa MBP-isoforms into N-terminal fragments of 10 and 8 kDa in the cortex. They also observed a similar degradation of the 17 and 14 kDa MBP-isoforms. Their identification was based on three matching tryptic peptides, two of which, DTGILDSIGR and TQDENPVVHFFK, were observed in our study. Ottens et al. [62] reported significant proteolysis of the 21.5, 14.2, and 18.5 kDa isoforms in a traumatic brain injury model. Six predicted calpain fragments of MBP were described including a 12.6 kDa fragment which may be related to the 12.4 kDa MBP fragment identified in our fraction Q3. Another EAE study [64] showed that the 18.5 and 14.2 kDa MBP isoforms were substantially degraded by calpain to 10 and 11 kDa fragments. Additionally, Matrix metalloproteinases (MMPs) are reported to play a significant role in the fragmentation of MBP and demyelination leading to multiple sclerosis and EAE [58]. The mechanisms of myelin breakdown in MS are not clearly established but degradation of MBP has been thought to be the initial step in the breakdown of myelin in demyelinating diseases. This hypothesis is supported by the degradation and loss of protein from MS plaque believed to be associated with increased activities of the proteolytic enzymes in demyelinating conditions such as MS and EAE.

## Conclusions

We have demonstrated that SELDI mass spectrometry can be employed to explore the proteome of a complex tissue (brain) and have obtained protein profiles of differentially expressed proteins from mitochondrially enriched protein fractions from experimental autoimmune encephalomyelitis (EAE) and control mouse brain. We have shown that appropriate homogenization protocols and protein fractionation using anion exchange beads can be employed to reduce sample complexity without introducing significant additional variation into the SELDI mass spectra beyond that inherent in the SELDI- MS method itself. Overall reproducibility is comparable to that reported by others for analyses performed on a single machine with a coefficient of variation less than 19%. Although, SELDI-MS coupled with principal component analysis and hierarchical cluster analysis provides protein patterns that can clearly distinguish the disease state from controls, the identification of individual differentially expressed proteins requires a separate purification of the proteins of interest by polyacrylamide electrophoresis prior to trypsin digestion and peptide mass fingerprint analysis. Unambiguous identification of differentially expressed proteins can be difficult if protein bands consist of several proteins with similar molecular weights. Our multi-dimensional use of subcellular fractionation and ion exchange fractionation prior to 1D PAGE was insufficient to resolve all proteins, although the use of a larger number of ion exchange fractions might have resolved more proteins. We have shown that myelin basic protein isoform 8 (MBP8) (14.2 kDa) levels are lower in EAE samples with advanced disease relative to controls, while an MBP fragment (12.4 kDa), likely due to calpain digestion, is increased in EAE brain tissue relative to controls. Although differential expression of MBP and its proteolytic fragments appears to be a consistent feature of EAE, we have shown that MBP is not found associated with mitochondria obtained from fresh tissue, and instead its appearance in mitochondrially enriched fractions used here is most likely due to tissue freezing and storage.

## Methods

### Materials

Reagents used for the preparation of all buffers were obtained from Sigma-Aldrich (St. Louis, MO).

### Brain tissue samples

EAE was induced in C57Bl/6 mice by subcutaneous injection of MOG_35-55_ as previously described [67]. EAE brains from mice at disease stage 1 and 3 and brains from control littermates were prepared at The University of Calgary and sent frozen to Kent State University. The reproducibility study was conducted with pooled tissue from six control mice brains.

### Subcellular fractionation of frozen tissue

Two tissue homogenization methods were tested. In the first method (M1), 60 μm tissue slices (approximately 250 mg) were cut and a Brinkmann blender was employed to homogenize the tissue. In the second method (M2), small blocks (approximately 250 mg) of brain tissue were cut and homogenized with a Teflon® pestle homogenizer. For both homogenization methods, frozen brain tissue was suspended in whole cell homogenization buffer (20 mM KCl, 3 mM MgCl_2_, 10 mM 4-(2-hydroxyethyl)-1-piperazine ethanesulfonic acid (HEPES), pH 7.9 0.5% NP-40, 5% glycerol, with protease inhibitors (P2714, Sigma-Aldrich, St. Louis, MO)). The cell homogenate was centrifuged at 1500 g for 10 minutes at 4°C. The supernatant was removed and centrifuged at 10,000 g for 15 minutes at 4°C. The supernatant was removed and stored at -80°C as the cytosolic fraction. The pellet containing the mitochondrially enriched fraction was washed twice in 20 mM phosphate buffered saline (PBS) pH 7.4. The mitochondria were then lysed in mitochondrial lysis buffer (50 mM Tris, 7M urea, 3% CHAPS with protease inhibitors) for 20 minutes at room temperature. The mitochondrial lysate was centrifuged at 10,000 g for 10 minutes at 4°C. A modified Lowry protein assay was used to determine the protein concentration. All samples were stored at -80°C until further analysis.

### Subcellular fractionation of fresh brain tissue

Brains were removed immediately from killed mice, weighed (around 300 mg) and immersed in ice cold mitochondria isolating buffer (MIB) (0.25 M sucrose, 0.5 mM potassium EDTA, 10 mM Tris-HCl pH 7.4). All glassware and equipment was kept cold (4°C). The brain tissue was homogenized in 4.0 mL of 12% percoll in MIB, with a Dounce homogenizer with 30 strokes. The homogenate (~3.5 mL) was then layered onto a previously poured 3.5 mL 26% percoll which itself had been poured over 3.5 mL of 40% percoll. The sample was centrifuged at 30,000 g for 1 minutes at 4°C. The top layer containing myelin and other cellular debris was carefully removed using a Pasteur pipette and discarded. The second layer containing the mitochondria was carefully removed and diluted 1:4 in cold MIB and centrifuge at 15,000 g for 10 minutes at 4°C. The resulting pellet was resuspended with 1 mL MIB and centrifuged once more at 15,000 g for 10 minutes. The pellet was then vortexed in mitochondria lysis buffer for 1 minute and incubated for 20 minutes at room temperature. The lysate was centrifuged at 10,000 g for 10 minutes at 4°C. The supernatant protein concentration was determined and the samples were stored at -80°C until further analysis.

### Ion exchange fractionation

Cytosolic and mitochondrially enriched fractions were further fractionated using ion exchange chromatography in a spin column format (UFC30HV00 column, UFC3000TB centrifuge tube, Millipore, Billerica, MA). Samples (100 μg each) were equilibrated with 50 μl of buffer A (9 M Urea, 2% CHAPS in 50 mM Tris HCl pH 9.0) for 20 minutes at room temperature. Buffer B (buffer A diluted 1 part to 8 parts with 50 mM Tris HCl, pH 9.0) was used to dilute samples to 200 μL. The quaternary ammonium anion exchange beads (Q ceramic HyperD F, Pall BioSera, New York, NY) were equilibrated with three changes of 200 μL of buffer B. Samples were loaded onto the column and mixed on an end-to-end mixer for 30 minutes at room temperature. Samples were centrifuged for 1 minute at 1000 g. This eluate was the flow-through fraction. The column was removed from the centrifuge tube and placed in the next centrifuge tube (Q1, pH 9). 400 μL of pH 9 elution buffer (50 mM Tris HCl pH 9.0, 0.1 OGP) was loaded onto the column and placed on the end-to-end mixer for 10 minutes and then centrifuged. This process was repeated for fraction Q2 (pH 7.0 elution buffer: 50 mM HEPES pH 7.0, 0.1% OGP), fraction Q3 (pH 5.0 elution buffer: 100 mM sodium acetate pH 5.0, 0.1% OGP), fraction Q4 (pH 4.0 elution buffer: 100 mM sodium acetate pH 4.0, 0.1% OGP), fraction Q5 (pH 3.0 elution buffer: 50 mM sodium acetate pH 3.0, 0.1% OGP), and fraction Q6 (organic wash: 33% isopropanol, 17% acetonitrile and 0.1% trifluroacetic acid). All fractions were stored at -80°C until use. Three fractions (Q1, Q2, and Q4) derived from the cytosolic fraction of pooled control mice brains were employed for the reproducibility study. The EAE study employed mitochondrial fractions (Q1- Q6), from individual EAE and control mice.

### ProteinChip array preparation

The samples obtained from ion exchange fractionation were analyzed using NP20 ProteinChip arrays (Ciphergen Biosystems, Fremont, CA). Each ProteinChip was prepared according to the Ciphergen ProteinChip Application guide (ProteinChip Applications Guide Volume 2). Briefly, 1 μL of each sample was applied directly to the ProteinChip array in a randomized manner. After air-drying for 3 minutes, all spots were treated with 1 μL applications of matrix (saturated sinapinic acid (SPA) in 0.5% trifluoroacetic acid, 50% acetonitrile) letting the spots dry between each application.

### Data acquisition and processing

Mass spectra were acquired using a model PBSIIc SELDI-TOF-MS (Ciphergen Biosystems, Fremont, CA). SELDI mass spectra were acquired with a laser intensity of 225 and a detector sensitivity of 8 for high molecular weight proteins (HMW protocol), and a laser intensity of 199 and a detector sensitivity of 9 for low molecular weight proteins (LMW protocol). Both high and low molecular weight spectra were acquired at a digitizer rate of 250 MHz in positive ion mode with a chamber vacuum of less than 5×10^-7^ torr, a source voltage of 20 kV and a detector voltage of 2,700 V. A total of 65 transients were averaged for each spectrum. Spectral processing (smoothing and baseline subtraction) was performed with ProteinChip 3.1 software. All spectra were calibrated externally using an All-in-1 peptide standard [porcine dynorphin, 2147.50 Da; bovine insulin β-chain, 3495.94 Da; and hirudin BHVK, 7033.61 Da], and an All-in-1 protein standard [bovine cytochrome c, 12230 Da; bovine carbonic anhydrase, 29024 kDa; and S. cerevisiae enolase, 46670 Da] (Ciphergen Biosystems, Fremont, CA). Peak intensities were normalized against the total ion current (TIC), excluding the mass range below 1500 Da, which is composed of strong signals from the SPA matrix. Because automatic peak selection by the Ciphergen Express software can miss features that appear as shoulders on large peaks, all spectra were subjected to manual peak-picking. All peaks that are present in at least 25% of the spectra and with SNR ≥ 3 were selected.

### Data analysis

Univariate (Mann-Whitney test) and multivariate statistical analysis (principal component analysis (PCA)) were performed using Ciphergen Express software. Analysis of variance (ANOVA) was employed to calculate the p-value. SELDI-MS peak intensity differences were considered significant when p ≤ 0.05. Hierarchical clustering was performed on all peaks that were present in at least 25% of spectra with SNR > 4.0 and heat maps were generated [49].

### Protein purification

For the EAE study, sample fractions containing differentially expressed proteins of interest were subjected to 1D electrophoresis on 16% acrylamide Tris-glycine gels. Briefly, each sample was concentrated in a Thermo Speed Vac vacuum concentrator, then dissolved in sample buffer (0.125 M Tris-HCl, 4% SDS, 40% glycerol, 0.1% bromophenol blue, pH 6.8, (Invitrogen)), followed by heating for 10 minutes at 75°C. 20 uL of sample mixture were loaded onto the appropriate lane in the gel and electrophoresed at 200Vfor 45 minutes. SeeBlue Plus2 (Invitrogen) pre-stained molecular weight standards were employed for calibration. The gel was stained with Coomassie Brilliant blue R 250 (0.25% Commassie blue, 40% methanol, 7% acetic acid) and were destained with destaining solution I (40% methanol, 7% acetic acid) for one hour, and with destaining solution II (7% acetic acid, 5% methanol) overnight.

### In-gel digestion

The desired protein bands were excised from the gel and subjected to in-gel trypsin digestion using a procedure employed at the Center for Proteomics and Bioinformatics, Case Western Reserve University. Gel plugs were covered with 25 mM ammonium bicarbonate (ABC) at room temperature for 10 minutes. The supernatant was replaced with 50% acetonitrile in 25 mM ABC for 10 minutes. These first two steps were repeated twice. Gel plugs were then dried in a Thermo Speed Vac vacuum concentrator. 10 mM dithiothreitol (DTT) in 25 mM ABC was added and the reaction allowed to proceed at 56°C for 45 minutes. The supernatant was then replaced with 55 m M iodoacetamide and the reaction proceeded at room temperature in the dark for 45 minutes. Trypsin solution (2 ng/μL trypsin in 25 mM ABC) was added and the digestion proceeded at 37°C overnight. Formic acid was used to quench the reaction. The digest solution containing the extracted peptides was stored at -4°C.

### Peptide mass fingerprinting

Proteins were identified by peptide mass fingerprinting and peptide sequencing using a Dionex Utimate 3000 capillary LC system on line with LTQ-Fourier Transform (FT) mass spectrometer (Thermo Electron Corp., Bremen, Germany) at the Center for Proteomics and Bioinformatics, Case Western Reserve University. The tandem mass spectra were annotated and peak list files were generated. The resulting peak list files were then used to interrogate sequences present in the indexed IPI mouse database with the Mascot algorithm (Matrix Science). A positive identification was accepted when a minimum of two peptide monoisotopic masses matched a particular protein with sequence coverage ≥10%, and low expectation value (p < 0.05).

### Western blotting

Protein samples were mixed with SDS-PAGE sample buffer (Invitrogen) followed by heating at 70°C for 10 minutes. Protein samples were separated by SDS-PAGE on NuPage 4-12% Bis-Tris mini gels (Invitogen) run at 200 V for 30 minutes, and then transferred to nitrocellulose paper at 45 V for 1 hour. The blot was blocked with 5% non-fat milk dissolved in 0.1% Tween-TBS buffer, and then incubated with antibody against Myelin Basic Protein overnight at 4°C. The blot was washed with 0.1% Tween-TBS buffer and incubated with the secondary antibody conjugated with horseradish peroxidase (HRP) for 1 hour. The blot was washed with 0.1% Tween-TBS buffer and visualized using chemiluminescence reagent (sc2048, Santa Cruz Biotechnology, Inc., Santa Cruz, CA). Images were made by exposing X-ray film (Fuji Super RX) to the blots. The images were analyzed using Image J software (NIH, Bethesda, MD).

### Preparation and use of fluor-labeled MBP

MBP was labeled with HiLyte Fluor 488 (Anaspec, Fremont, CA) according to the manufacturer’s instructions. Briefly, 100 μg of MBP was dialyzed overnight against 50 mM PBS, pH 7.4 at 4°C. Fluor was suspended in DMSO to generate 10 μL of a 2 mM solution. Protein labeling was conducted at a 7.5:1 ratio of fluor to protein at room temperature for 45 minutes. The dye-conjugated protein was washed with 50 mM PBS, pH 7.4 and separated from unreacted dye by centrifugation in a spin column.

### Sample preparation

Whole brains were harvested from euthanized pregnant wistar rats and stored at -80°C. Brains were homogenized in parallel with and without 500 μL of fluorescent MBP added to 1.5 mL of general lysis buffer (20 mM KCl, 3 mM MgCl2, 10 mM 4-(2-hydroxyethyl)-1-piperazineethanesulfonic acid (HEPES) pH 7.9, 0.5% NP-40, 5% glycerol with protease inhibitors (P2714, Sigma-Aldrich, St. Louis, MO using a Wheaton homogenizer with a Teflon® pestle. in thirty strokes. The homogenate was centrifuged for 10 minutes at 500g at 4°C. The supernatant was removed and centrifuged at 10,000 g for 30 minutes at 4°C. The pellet containing the mitochondrially enriched fractions was further purified by washing once in 20 mM phosphate buffered saline (PBS), pH 7.4. The mitochondrial fractions were stored at 4°C.

### Fluorescent spectroscopy

Emission spectra were acquired on a Cary Eclipse spectrofluorimeter. The fluorescence intensity at 523 nm was employed to compare the levels of fluor-labelled MBP observed in each sub-cellular fraction.

### Ethical approval

All mice, from which EAE and control brain tissue was obtained, were handled in accordance with the policies outlined by the Canadian Council for Animal Care and the University of Calgary. All other animals were handled according to guidelines of the Institutional Animal Care and Use Committee at Kent State University outlined in protocols 338 and 350.

## Competing interests

The authors declare no competing interests.

## Authors’ contributions

SA collected and analyzed the data for the SELDI-MS reproducibility study and EAE protein profile study and contributed to the interpretation of the results and the writing; LB performed the fluorescence studies of MBP contamination, SL performed the Western blot analysis of MBP contamination in fresh and frozen tissue, EF and JM contributed to the study design and the interpretation of the results, RBG contributed to the study design, interpretation of the results and the writing. All authors read and approved the final manuscript.
